# Beijing/W Genotype *Mycobacterium tuberculosis* and Drug Resistance

**DOI:** 10.3201/eid1205.050400

**Published:** 2006-05

**Authors:** European Concerted Action on New Generation Genetic Markers and Techniques for the Epidemiology and Control of Tuberculosis

**Affiliations:** * RIVM, Bilthoven, the Netherlands

**Keywords:** tuberculosis, molecular epidemiology, drug resistance, Beijing strain, research

## Abstract

Beijing/W genotype *Mycobacterium tuberculosis* is widespread, may be increasing, and may have a predilection for drug resistance. Individual-level data on >29,000 patients from 49 studies in 35 countries were combined to assess the Beijing genotype’s prevalence worldwide, trends over time and with age, and associations with drug resistance. We found 4 patterns for Beijing/W genotype tuberculosis (TB): 1) endemic, not associated with drug resistance (high level in most of East Asia, lower level in parts of the United States); 2) epidemic, associated with drug resistance (high level in Cuba, the former Soviet Union, Vietnam, and South Africa, lower level in parts of Western Europe); 3) epidemic but drug sensitive (Malawi, Argentina); and 4) very low level or absent (parts of Europe, Africa). This study confirms that Beijing/W genotype TB is an emerging pathogen in several areas and a predominant endemic strain in others; it is frequently associated with drug resistance.

## Abstract 2

The genotype, endemic in some areas and emerging in others, may be associated with drug-resistance.

The *Mycobacterium tuberculosis* genotype family known as "Beijing/W," "W-Beijing," or "Beijing" is widespread (*1**–**3*). Described in 1995 as the prevalent genotype in East Asia (*4*), >80% of strains from the Beijing area were of this type. The multidrug-resistant W strain is a member of the family. We use "Beijing" for the whole genotype family.

Researchers are concerned that the Beijing genotype may have a predilection for developing drug resistance (*5*) and may be spreading worldwide, perhaps as a result of increased virulence (*6*). A systematic review of the published literature in 2002 concluded that although Beijing genotype tuberculosis (TB) was widespread, associations with drug resistance varied, and little information on time trends was available (*2*).

The review highlighted the problems of relying on published literature: varying strain definitions; reporting bias; and limited information on selection criteria, population subgroups, age groups, or time trends. As part of the European Concerted Action on New Generation Genetic Markers and Techniques for the Epidemiology and Control of Tuberculosis, we have combined available datasets, using a common strain definition and individual-level data.

## Methods

Studies for inclusion were identified from the systematic review and from contacting members of the European Concerted Action and authors of relevant articles published since the review. We aimed to include as many studies as possible in which the proportion of TB caused by the Beijing genotype could be ascertained in an unbiased way. Studies could represent all or random samples of patients in an area, hospital, or laboratory. Studies limited to outbreaks, drug-resistant isolates, or of <30 patients were excluded. A study description and individual patient data that included at least the year the case was diagnosed and the genotype were required.

### Strain Classification

Three methods identify Beijing genotype strains: spoligotyping (*7*), IS*6110* restriction fragment length polymorphism (RFLP) (*8*), and region A RFLP (*9*). The typical Beijing spoligotype shows hybridization to spacers 35–43. Beijing-like patterns with <9 spacers (but not solely spacers 37–38, which represents *M. microti*), were included (*10*).

By using IS*6110* RFLP, fingerprints are compared to 19 patterns representative of the Beijing genotype (https://hypocrates.rivm.nl/bnwww/index.html). With standard techniques, allowing 1% position tolerance and classifying all matches >80% as Beijing, these patterns have 96%–100% sensitivity and 98%–100% specificity to detect Beijing strains, taking spoligotyping as the accepted standard (*10*). Sensitivity is increased by spoligotyping strains with RFLP patterns that match 75%–80% to the reference strains. The third technique uses a characteristic IS*6110* insertion in region A. This method has 100% sensitivity and 98% specificity compared with spoligotyping (*10*).

### Analysis

The proportion of Beijing genotype strains in each study was calculated overall and after excluding immigrants. The proportion of Beijing genotype in immigrants was examined by place of birth. Time trends were examined directly and by examining trends with age; an association with younger age groups would suggest that the proportion of TB attributable to the Beijing genotype was increasing. Associations with drug resistance were examined, after immigrants were excluded, with and without excluding patients with previous TB. For pooled analyses, heterogeneity in the associations between studies was examined, and the results presented are adjusted for study.

## Results

Data were received from 49 studies representing 29,259 TB patients in 35 countries, including 11 studies from the systematic review (*2*); other studies in the review had no individual patient data available, used nonstandard case definitions, or researchers declined to participate. Other studies were contributed by members or contacts of the Concerted Action or were identified from subsequently published studies. Details of all included studies are shown in Table A1.

The proportion of tuberculosis due to the Beijing genotype in the included studies is shown in Table A2.

Overall, 9.9% had the Beijing genotype. In Western Europe and the Czech Republic, the proportion was low: <6% of cases among nonimmigrants. In sub-Saharan Africa, the proportion was low except in Cape Town, South Africa. In Latin America, data were only available from Argentina and Brazil; both studies found <1% of TB cases were caused by Beijing genotype. In North America and the Caribbean, the proportion was higher (8%–14%). In the former Soviet Union the proportion was high: 45%–56% in Russia and 29% in Estonia. The proportion was low in India (1%), higher in Bangladesh (7%), and increased further east: >50% in many parts of Southeast and East Asia.

Analyses by region of birth showed similar patterns (Table 1). Beijing genotype strains were rare (0.5%) among immigrants from Eastern Europe other than the former Soviet Union; most came from the former Yugoslavia. The Beijing genotype was much less common among immigrants from the Indian subcontinent (3.4%) than among those from Southeast Asia (19%) or East Asia (58%). Beijing genotype strains were uncommon among immigrants from North Africa (3.0%), the Middle East (5.2%), and sub-Saharan Africa (2.2%, including 50 [2.1%] of 2,427 persons from Somalia). Among Middle Eastern immigrants, Beijing genotype was found in 6 (1%) of 620 persons from Turkey but in 8 (9.9%) of 81 from Afghanistan.

**Table 1:** Proportion of tuberculosis patients due to the Beijing genotype by region of birth

Region	All patients, Beijing/total (%)	Immigrants only, Beijing/total (%)
Western Europe	272/9,496 (2.9)	10/353 (2.8)
Central and Eastern Europe	4/780 (0.5)	3/562 (0.5)
Former Soviet Union	244/590 (41.4)	25/106 (23.6)
Middle East	62/1,165 (5.3)	56/1,084 (5.2)
North Africa	30/991 (3.0)	30/991 (3.0)
Sub-Saharan Africa	275/6,816 (4.0)	86/3,881 (2.2)
Indian subcontinent	46/1,291 (3.6)	38/1,111 (3.4)
Southeast Asia	711/2,192 (32.5)	154/811 (19.0)
East Asia	1,032/1,712 (60.3)	213/370 (57.6)
Latin America	29/1,421 (2.0)	21/457 (4.6)
Caribbean	31/320 (9.7)	5/109 (4.6)
North America	28/275 (10.2)	1/15 (6.7)
Australasia	1/4 (25.0)	1/4 (25.0)

### Time Trends

Time trends were analyzed among nonimmigrants within individual studies with >3 years of data (Table 2). (Studies from France, Iran, Thailand, Vietnam, and Spain are excluded because of small numbers in some years or absence of Beijing genotype strains).

**Table 2:** Trends in proportion of tuberculosis cases due to the Beijing genotype over time among nonimmigrant populations*

Study	Period	Earlier period,† Beijing/total (%)	Later period,† Beijing/total (%)	OR (95% CI) for change/y	p for linear trend by y
Western Austria	1993–2004	2/363 (0.6)	5/310 (1.6)	1.2 (0.9–1.5)	0.2
Denmark	1992–2001	7/885 (0.8)	10/774 (1.3)	1.1 (0.9–1.3)	0.4
Finland	2000–2002	2/414 (0.5)	11/705 (1.6)	1.7 (0.9–3.5)	0.1
The Netherlands	1993–2002	91/1,862 (4.9)	111/1,607 (6.9)	1.1 (1.0–1.1)	0.004
Western Sweden	1999–2002	0/34 (0.0)	3/43 (7.0)	3.1 (0.6–15)	0.2
London, UK	1995–1997	9/200 (4.5)	1/73 (1.4)	0.7 (0.3–1.8)	0.4
St. Petersburg, Russia	1999–2001	66/120 (55.0)	67/116 (57.8)	1.0 (0.7–1.3)	0.9
Cape Town, South Africa	1992–1998	60/473 (12.7)	80/374 (21.4)	1.2 (1.1–1.3)	<0.001
Karonga, Malawi‡	1996–2003	12/460 (2.6)	32/570 (5.6)	1.2 (1.0–1.4)	0.03
San Francisco, USA	1998–2000	6/50 (12.0)	6/59 (10.2)	1.0 (0.5–2.1)	1.0
Buenos Aires, Argentina	1998–2001	1/188 (0.53)	4/424 (0.94)	1.0 (0.4–2.3)	1.0
São Paulo, Brazil	2000–2002	2/268 (0.75)	1/114 (0.88)	1.0 (0.2–4.3)	1.0
Okayama, Japan	2000–2002	42/56 (75.0)	61/86 (70.9)	0.8 (0.5–1.3)	0.4

*OR, odds ratio; CI, confidence interval. †For each study, the period was split into 2 parts, earlier and later. ‡Includes immigrants from neighboring countries.

All Western European sites except London showed a slight increase in Beijing strains over time, but this finding was only significant in the Netherlands. Combining data for Western Europe, the odds ratio (OR), adjusted for study, for having the Beijing genotype in the later period compared to the earlier period was 1.5 (95% confidence interval [CI] 1.2–1.9). This figure was unchanged after adjusting for age. The trend was similar after excluding the Netherlands (adjusted OR 1.7, 95% CI 0.96–3.1).

In St. Petersburg, Okayama, Buenos Aires, São Paulo, and San Francisco, no significant change occurred over time, but the studies only covered a few years. In Cape Town and Malawi, significant increases occurred over time and were unchanged after adjusting for age.

### Trends with Age

Trends with age for studies with >3 cases of Beijing genotype TB among nonimmigrants are summarized in Table 3. Most Western European studies found the highest proportion of Beijing genotype TB in the youngest age groups. Overall, for Western Europe, compared to those age >50 years, the OR, adjusted for study, of having the Beijing genotype was 1.2 (0.87–1.6) for those 30–49 years of age, and 2.4 (95% CI 1.8–3.3) for those <30 years of age, p_trend_<0.001. Excluding the Netherlands, the trend was stronger: adjusted OR 2.2 (95% CI 1.1–4.2) for those 30–49 years of age and 3.9 (95% CI 1.9–7.9) for those <30 years of age, p_trend_<0.001.

**Table 3:** Proportion of tuberculosis cases caused by the Beijing genotype by age group of patient*

Study	Age <30 y, Beijing/total (%)	Age 30–49 y, Beijing/total (%)	Age >50 y, Beijing/total (%)	p for trend
Western Europe				
	Western Austria	2/89 (2.3)	45/214 (1.9)	1/370 (0.3)	0.05
	Denmark	4/210 (1.9)	6/623 (1.0)	7/826 (0.9)	0.3
	Finland	2/35 (5.7)	5/128 (3.9)	6/931 (0.6)	0.002
	The Netherlands	70/703 (10.0)	47/993 (4.7)	85/1773 (4.8)	<0.001
	Western Sweden	1/5 (20.0)	1/7 (14.3)	1/65 (1.5)	0.05
	United Kingdom				
	Inner London	1/41 (2.4)	2/67 (3.0)	2/55 (3.6)	0.7
	London	6/86 (7.0)	1/104 (1.0)	3/83 (3.7)	0.2
Eastern Europe				
	Estonia	14/43 (32.6)	25/96 (26.0)	15/52 (28.9)	0.7
	Russia				
	St. Petersburg	74/112 (66.1)	61/111 (55.0)	19/45 (42.2)	0.02
	Archangel†	13/25 (52.0)	32/77 (41.6)	8/16 (50.0)	0.8
Middle East				
	Iran	2/20 (10.0)	2/25 (8.0)	1/26 (3.9)	0.4
Sub-Saharan Africa				
	Malawi‡	19/341 (5.6)	21/522 (4.0)	4/167 (2.4)	0.08
	South Africa: Cape Town	51/299 (17.1)	77/434 (17.7)	11/111 (9.9)	0.2
	Zimbabwe: Harare	3/94 (3.2)	1/102 (1.0)	0/16 (0.0)	0.2
North America				
	United States				
	New Jersey	1/18 (5.6)	11/62 (17.7)	3/71 (4.2)	0.2
	San Francisco	1/21 (4.8)	7/58 (12.1)	4/30 (13.3)	0.4
Caribbean				
	Cuba				
	Not Havana	10/48 (20.8)	6/42 (14.3)	6/70 (8.6)	0.06
	Havana	1/11 (9.1)	2/21 (9.5)	1/19 (5.3)	0.7
Latin America				
	Argentina: Buenos Aires	5/255 (2.0)	0/224 (0.0)	0/103 (0.0)	0.05
	Brazil: São Paulo	3/144 (2.1)	0/187 (0.0)	0/51 (0.0)	0.1
Indian subcontinent				
	Bangladesh†	3/20 (15.0)	4/42 (9.5)	0/35 (0.0)	0.03
Southeast Asia				
	Indonesia: Jakarta	13/45 (28.9)	14/33 (42.4)	5/12 (41.7)	0.2
	Malaysia	17/93 (18.3)	20/129 (15.5)	25/162 (15.4)	0.6
	Thailand: Bangkok	33/64 (51.6)	41/88 (46.6)	24/52 (46.2)	0.5
	Vietnam				
	Hanoi†	11/15 (73.3)	17/26 (65.4)	9/23 (39.1)	0.03
	Ho Chi Minh City†	94/147 (64.0)	134/265 (50.6)	35/87 (40.2)	<0.001
	Ho Chi Minh City	13/21 (61.9)	17/40 (42.5)	4/14 (28.6)	0.04
	Tien Giang	4/7 (57.1)	11/27 (40.7)	13/26 (50.0)	1.0
East Asia				
	China				
	Shanghai and other areas†	5/5 (100.0)	10/14 (71.4)	16/24 (66.7)	0.2
	Henan	10/19 (52.6)	7/9 (77.8)	16/21 (76.2)	0.2
	Hong Kong†	95/151 (62.9)	149/197 (75.6)	112/152 (73.7)	0.04
	Japan: Okayama	9/12 (75.0)	19/25 (76.0)	75/105 (71.4)	0.7
	Mongolia	50/95 (52.6)	42/63 (66.7)	5/10 (50.0)	0.3
	Taiwan†	25/47 (53.2)	36/83 (43.4)	126/291 (43.3)	0.3

*Studies with >3 cases of Beijing genotype tuberculosis in nonimmigrants included. †Immigration status not known. ‡Immigrants from neighboring countries included.

In Russia and Estonia, Beijing genotype strains were more common in younger patients, and the trend was significant in St. Petersburg (p = 0.02). Overall, compared to those >50 years of age, the study-adjusted OR was 1.1 (95% CI 0.70–1.8) for those 30–49 years of age and 1.7 (95% CI 1.1–2.9) for those <30 years of age, p_trend_ = 0.02.

The African studies that found any Beijing strains noted a higher proportion in younger persons than in older persons. This difference was not significant in individual studies but was when studies were combined: adjusted OR, 1.9 (95% CI 1.1–3.4) for those 30–49 years of age and 2.1 (95% CI 1.2–3.7) for those <30 years of age, compared to those >50 years of age, p_trend_ = 0.03.

Among nonimmigrants in US studies, no significant trend occurred with age, either individually or overall. In Cuba, Beijing genotypes were more common in younger persons than in older persons in the larger study and overall (p_trend_ = 0.06). In Buenos Aires and São Paulo, all Beijing genotype–infected patients were <30 years of age (p = 0.002).

Most Asian studies showed no association with age, but trends were seen in Bangladesh, Vietnam, and Hong Kong. In Vietnam, Beijing genotype was more common in younger patients in all 4 studies: overall, compared to those >50 years of age, the study-adjusted OR was 1.5 (95% CI 1.0–2.2) for those 30–49 years of age and 2.7 (95% CI 1.7–4.2) for those <30 years of age, p_trend_<0.001. In Hong Kong, Beijing genotypes were least common in patients <30 years of age.

### Drug Resistance

Studies with drug resistance data for all or most patients and with >3 Beijing genotype TB patients among nonimmigrants are summarized in Table A3. In the Western European studies, with the exception of inner London, resistance was more common among Beijing genotype strains than among other strains. Beijing genotype was significantly associated with resistance in Denmark (rifampin and ethambutol), Finland (rifampin and streptomycin), and the Netherlands (streptomycin). Overall, the study-adjusted OR for the association of Beijing genotype and resistance among nonimmigrants in Western Europe was 1.8 (95% CI 1.2–2.7) for any drug, 1.7 (95% CI 0.95–2.9) for isoniazid, 4.0 (95% CI 1.4–11.9) for rifampin, 2.3 (95% CI 1.4–3.7) for streptomycin, 3.0 (95% CI 0.38–23.2) for ethambutol, and 4.2 (95% CI 1.2–14.7) for multidrug resistance (i.e., resistance to at least isoniazid and rifampin). Of the Western European studies, only those from Denmark, Hamburg, the Netherlands, and London had data on previous treatment. After patients who had previously received treatment were excluded, the associations in the Netherlands and Denmark persisted, and the adjusted combined ORs were similar to those overall but with wider CIs (e.g., 1.6, 95% CI 1.0–2.6 for any drug resistance).

In Russia and Estonia, Beijing genotype was strongly associated with resistance to all tested drugs. None of the patients in Estonia had been previously treated. In the Archangel Oblast, the association persisted after previously treated patients were excluded, but in St. Petersburg only the association with isoniazid resistance remained significant. In Cuba, Beijing genotype was associated with streptomycin resistance in both studies, and this association persisted after previously treated patients were excluded.

In Malawi and Zimbabwe, none of the Beijing genotype isolates was drug resistant. In Cape Town, 14 (35%) of the 40 Beijing isolates that were tested were drug resistant, but the resistance of most Beijing isolates and of the other isolates was unknown.

In the Asian studies, only those in Bangladesh, Vietnam, and Taiwan found more drug resistance in Beijing genotype strains. In Bangladesh, 99% of the patients had previously received treatment for TB. In Vietnam, the results were little changed by excluding the few previously treated patients. In Taiwan, previous treatment was unknown. Two studies found that Beijing genotypes were less commonly drug resistant. In China, Beijing genotypes were less likely to exhibit ethambutol resistance; no information was available on previous treatment. In Malaysia, among patients without previous treatment, 1 (2%) of 48 isolates from patients with the Beijing genotype and 33 (13%) of 252 isolates from patients with other genotypes were resistant to any drugs (p = 0.03).

### Other Associations

In most studies, the proportion of nonimmigrants with the Beijing genotype was similar for men and women. In Japan, the proportion was higher among men, and in Malawi, it was higher among women. Only 23 studies had data on HIV status in nonimmigrants, and of these, 13 found no Beijing genotype, no HIV-positive patients, or information was lacking on HIV status of the patients with Beijing genotype. In the 10 remaining studies (inner London, Lyon, the Netherlands, Tuscany, San Francisco, Cuba [both studies], Buenos Aires, Malawi, and Ho Chi Minh City), no association was found between HIV status and Beijing genotype.

No significant association was found between strain type and site of tuberculosis (pulmonary or extrapulmonary) in any of the 20 studies in which this information was available and both types of tuberculosis were included. In Cuba, outside Havana, and in the Archangel Oblast patients with recurrent TB were more likely than patients in their first episode of disease to have the Beijing genotype, but these associations were lost after adjusting for drug resistance. No associations with previous TB were found in any of the other 17 studies for which information was available, but the numbers of recurrent cases were often small.

## Discussion

In this study, we have brought together published and unpublished data to document the spread of Beijing genotype tuberculosis worldwide. Little information was available from many countries including most of the Americas, Eastern Europe, North Africa, the Middle East, and Australasia. All eligible studies were requested, whether Beijing genotypes were found or not, and within the included studies, the proportion with Beijing genotype should be representative of those settings. The individual-level data allowed comparable analyses in all sites and pooled analysis within regions. This study complements the spoligotype database (*3*), which includes only studies that used spoligotyping and is more inclusive and less detailed epidemiologically. The database shows a similar global distribution of the Beijing genotype to that described here.

The proportion of TB attributable to the Beijing genotype is variable: high in Asia, apart from the Indian subcontinent, increasing further east; low in parts of Africa, Latin America, and Western Europe; intermediate in the United States and Cuba; low in Eastern Europe (other than the former Soviet Union); low in the Middle East (including <1% in a recent study from Tehran [11]). In Western Europe, Beijing genotype is more common among immigrant TB patients than among indigenous patients. The proportion of Beijing genotype TB among nonimmigrants may reflect the importance of immigrants to the total TB prevalence in these countries as well as the origin of these immigrants. Immigrants accounted for >50% of TB cases in London, the Netherlands, France, Denmark, Sweden, and Hamburg, compared to 25% of cases in Italy, 24% in Austria, 8% in Finland, and 4% in Spain.

Using information from time and age group trends, we found that an increasing proportion of TB is due to Beijing genotype strains in Western Europe, southern Africa, and the former Soviet Union. We found little evidence of increase in Asia, except in Vietnam and Bangladesh.

Strong associations with drug resistance have been found in the former Soviet Union, Cuba, and Vietnam. The combined data for Western Europe suggest an association there. No association was found in a large study in Malawi or in most of the Asian studies.

When the data on trends and drug resistance presented here and from other studies are combined, the results suggest that the distribution of Beijing genotype TB has several patterns (Figure). The Beijing genotype probably originated in the Beijing region of China (*1**,**4*); it was found in 90% of stored biopsy specimens in the 1950s, and this proportion has not changed over time (*12*). Beijing strains appear to have spread and become established as the predominant *M*. *tuberculosis* genotype in much of East and Southeast Asia, so little evidence of increase was found. In these areas, the Beijing genotype appears to be endemic and not associated with drug resistance (pattern 1).

**Figure:**
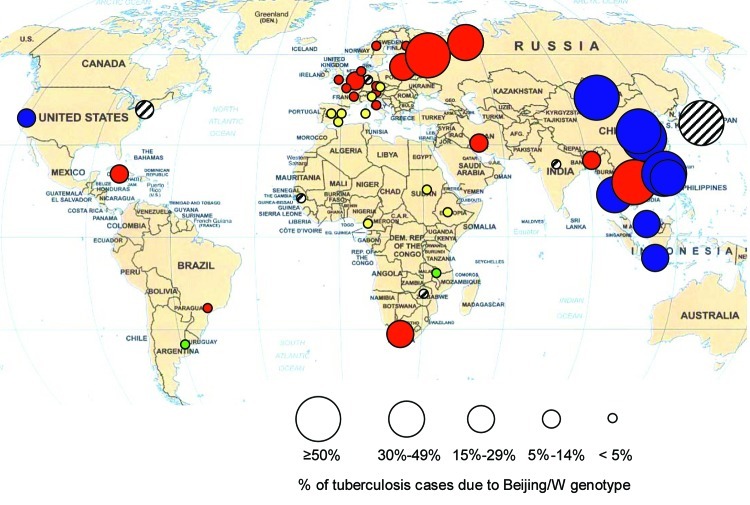
Distribution of Beijing genotype tuberculosis (TB). Size of circle indicates percentage of TB cases due to Beijing genotype; color in circle indicates drug sensitivity and distribution trend. Blue, pattern 1 (stable, no association with drug resistance); red, pattern 2 (increasing, associated with drug resistance); green, pattern 3 (increasing, drug sensitive); yellow, pattern 4 (absent); striped, trend or association with drug resistance not known.

In certain areas, including the former Soviet Union (*13*), Cuba, and Cape Town, epidemic spread was found, which was associated with drug resistance (pattern 2). Vietnam and Bangladesh follow this pattern, unlike most other Asian countries. Recent Indian studies suggest that India may also fit pattern 2 (*14**,**15*). In Taiwan, the association with drug resistance was not confirmed in a larger sample in 2003 (unpub. data), which suggests that it follows pattern 1. In parts of Western Europe, although the Beijing genotype remains uncommon, it appears to be increasing and is associated with drug resistance (pattern 2).

In the United States, the pattern is mixed. Nonimmigrant patients in San Francisco fit pattern 1: no association with drug resistance and no evidence of time trends. In this area, most Beijing isolates came from Asian immigrants, among whom no association was found between Beijing genotype and drug resistance. In the New Jersey study, no data on drug resistance were available, but a previous study in this area found that most Beijing isolates from nonimmigrants were pansusceptible (*1**,**16*). The age distribution does not suggest recent increase, which fits pattern 1. In contrast, the spread of the multidrug-resistant W strain in New York and beyond during the 1990s has been well documented (*17**–**19*). Other published studies from the United States confirm that the Beijing genotype is widespread but do not report drug resistance or trends (*20**–**23*).

In Malawi, an increase in the Beijing genotype over time was documented, but with drug sensitive strains (pattern 3). Argentina may fit this pattern, and spread of drug-sensitive Beijing genotype TB has been described in Gran Canaria (*24*). The final pattern (pattern 4) is of very low level or absent Beijing genotypes, as seen in parts of Africa and Europe.

The wide distribution of the Beijing genotype could be attributable to a founder effect or random drift, though these mechanisms would be unlikely to account for recent increases in multiple settings. The distribution could reflect particular stability of the genetic markers used to identify the genotype. High levels and epidemic spread may suggest that it transmits more easily or is more virulent than other strains. In vitro and animal studies have suggested increased multiplication or virulence for some Beijing strains (*6**,**25*) but not others (*26*). In Vietnam, the Beijing genotype was associated with treatment failure and relapse (*27*), but we found no such association. In Indonesia, patients with the Beijing genotype had a similar clinical picture to other TB patients for almost all parameters studied (*28*). In the Netherlands, the appearance on chest radiograph was similar for patients infected with Beijing genotype and for other TB patients (*29*). In Malawi, the Beijing genotype was not associated with death or transmissibility (*30*).

External factors may select for Beijing strains. In the former Soviet Union and the United States, spread has been associated with prisons and with high rates of drug resistance (*13**,**17**,**31**,**32*). In Mongolia, data were also available from prisoners. They had a higher proportion of Beijing genotype than did other patients, 46 (82%) of 56 compared to 97 (58%) of 168, p = 0.001, and a higher prevalence of drug resistance. Population movements (*33*), for example, from the former Soviet Union into Western Europe and through Afghanistan, may account for spread, recent increases, and the association with drug resistance (*34*).

Beijing genotypes may have a particular propensity to acquire drug resistance. Mutations in putative mutator genes have been found in Beijing genotypes, which suggests adaptability (*5*), but no increase in the rate of acquisition of resistance to rifampicin was found in in vitro studies (*35*). Once established, resistance could encourage spread if it delays effective treatment. Although the fitness of resistant strains is slightly reduced, this may be less marked for Beijing strains (*36*).

## Conclusion

This study has confirmed that Beijing genotype *M. tuberculosis* is an emerging infection in many parts of the world and is a highly endemic pathogen in other areas. Its association with drug resistance, sometimes at high levels, in a number of settings, underlines its importance. The reasons for its apparent success are not well understood but may depend on human population movements as well as on any intrinsic factors.

## Appendix

**Table A1:** Included studies on Beijing/W genotype *Mycobacterium tuberculosis* and drug resistance*

Country		Period	Source	Type of TB	No previous TB %	Typing method	Reference†
Western Europe							
	Austria: western		1993–2004	Laboratory receiving from nearly all hospitals in area	Pulm/Extra	?	Spoligo on all	
	Denmark: entire		1992–2001	Central laboratory for entire country	Pulm/Extra	97	RFLP, spoligo	(37)
	Finland: entire		2000–2002	Central laboratory for entire country	Pulm/Extra	?	RFLP, spoligo	(38)
	France							
		Paris area	1995	All patients from 10 hospitals	Pulm/Extra	88	RFLP, spoligo	(*39*)
Paris		2001–2003	Patients at 1 hospital	Pulm/Extra	?	Spoligo on all		
Lyon		2003	Central laboratory for area	Pulm/Extra	77	Spoligo on all		
Germany: Hamburg		2001	Notified patients in area	?	99	RFLP, spoligo	(*40*)
Italy							
		Verona	1996–1997	Reference laboratory for area	Pulm/Extra	?	Spoligo on all	(*41*)
		Sardinia	1997–1998	Patients at 1 hospital	Pulm/Extra	73	RFLP, spoligo	
		Tuscany	2002	All patients in area	Pulm/Extra	83	RFLP, spoligo	(*42*)
	Netherlands: entire		1993–2002	All patients in area	Pulm/Extra	96	RFLP, spoligo	(*43*)
	Spain							
		Elche	1993–1999	Sample of patients in area	Pulm/Extra	90	RFLP, spoligo	(*44*)
		Madrid	1992–2001	All patients in area	Pulm/Extra	71	RFLP, spoligo	
		Zaragoza	1993–1995	All patients in area	Pulm/Extra	?	RFLP, spoligo	(*45*)
Sweden: western		1999–2002	Laboratory for all patients in area	Pulm/Extra	87	Spoligo on all	(*46*)
United Kingdom							
		Inner London	1993	Laboratories for all patients in area	Pulm/Extra	89	Spoligo on all	(*47*)
		London	1995–1997	Laboratories for all patients in area	Pulm/Extra	88	RFLP	(*48*)
		Central and Eastern Europe							
		Czech Republic: Prague and South Moravia		1998	Laboratories for all patients in areas	Pulm/Extra	?	Spoligo on all	(*49*)
	Estonia: entire		1994	All new pulmonary TB patients in area	Pulm	100	RFLP, spoligo	(*50*)
	Russia							
		St. Petersburg	1999–2002	All patients in area with data	Pulm/Extra	59	RFLP, spoligo	(*51**,**52*)
Archangel		1998–1999	All patients at TB clinic	Pulm	75	Spoligo on all	(*53*)
North Africa and Middle East							
	Iran: several areas		1995–1997	All smear-positive patients from Shiraz plus random others	Pulm/Extra	79	RFLP, spoligo	(54)
Sub-Saharan Africa							
	Cameroon: western region		1997–1998	All patients from all hospitals in region	Pulm	79	Spoligo on all	(55,56)
	Ethiopia: Addis Ababa		1996	Patients at 1 hospital	Pulm	85	RFLP, spoligo	(*57*)
	Guinea Bissau: Bissau		1989–1993	All patients in area	Pulm	100	RFLP, spoligo	(*58*)
	Malawi: Karonga District		1996–2003	All patients in area	Pulm/Extra	93	RFLP, spoligo	(*59*)
	South Africa: Cape Town		1992–1998	All patients from 2 clinics	Pulm/Extra	91	RFLP, spoligo	(*60*)
	Sudan: Khartoum		1998–1999	Patients from 2 clinics	Pulm	54	Spoligo on all	(*61*)
	Zimbabwe: Harare		1997	All patients from 1 hospital	Pulm	93	Spoligo on all	(*62*)
North America							
	United States							
		New Jersey	1999	All patients in area	Pulm/Extra	99	RFLP, spoligo, region A	(*63*)
San Francisco		1998–2000	All patients in area	Pulm/Extra	92	RFLP	(*64*)
Caribbean							
	Cuba							
		Outside Havana	1994–1995	Isolates sent from regional laboratories	Pulm	94	RFLP, spoligo	(*65*)
Havana		1997–1998	Isolates sent to reference laboratory	Pulm	86	RFLP	(*66*)
Latin America							
	Argentina: Buenos Aires		1998–2001	All patients in 1 hospital	Pulm/Extra	82	Spoligo on all	
	Brazil: São Paulo		2000–2002	All patients in area	Pulm	72	RFLP	
Indian subcontinent							
	India: Delhi		1995–1996	Patients from 2 male wards + clinic	?	57	RFLP, spoligo	(*67*)
	Bangladesh: Mymensingh		2001–2003	Hospitalized patients, 3 hospitals	Pulm	1	Spoligo on all	(*68*)
Southeast Asia							
	Indonesia: Jakarta		1998–1999	All patients from outpatient TB clinic	Pulm	75	RFLP,spoligo	(*69*)
	Malaysia: entire		1993–1994	Random sample from reference laboratory	Pulm/Extra	89	RFLP	(*70*)
	Thailand: Bangkok		1998–2000	All patients in single hospital	Pulm/Extra		Spoligo on all	(*71*)
	Vietnam							
		Hanoi, Ho Chi Minh City	1998–1999	All new smear-positive patients at 2 centers	Pulm	?	Spoligo on all	(*72*)
Ho Chi Minh City		1998–2000	HIV stratified random sample (1/3 HIV positive)	Pulm	100	RFLP, spoligo	
Tiet Giang Province		2003	Age stratified sample from laboratory (1/2 patients <40 y)	Pulm	93	RFLP, spoligo	
East Asia							
		China							
		Shanghai and other areas	1994–1995	Random sample from laboratory	?	?	Spoligo on all	
		Henan	2001–2002	All patients in 1 hospital (severe cases)	Pulm	87	RFLP	
	Hong Kong: Hong Kong		1998–1999	Random sample from laboratory	?	100	Spoligo on all	(*73*)
	Japan: Okayama		2000–2002	All patients in area with data	Pulm/Extra	99	RFLP, spoligo	(*74*)
	Mongolia: entire		1998–1999	Random sample of patients in area	Pulm	100	Spoligo on all	(*75*)
	Taiwan: entire		2002	Random sample from laboratory	Pulm/Extra	?	Spoligo on all	(*76*)

*TB, tuberculosis; Pulm, pulmonary; extra, extrapulmonary; spoligo, spoligotyping; RFLP, restriction fragment length polymorphism, IS*6110* fingerprinting; region A, characteristic insertion in region A. †The references given describe the studies from which the data came. In many cases, the analysis of Beijing strains has been carried out specifically for this collaborative study. The key contacts who contributed the data are listed as follows: Austria: Wolfgang Prodinger (Medizinische Universität Innsbruck); Denmark: Troels Lillebaek (Statens Serum Institut, Copenhagen); Finland: Hanna Soini, Petri Ruutu, (National Public Health Institute, Helsinki); France: Cristina Gutierrez, Veronique Vincent (Institut Pasteur, Paris); Beate Heym, Veronique Friocourt (Hôpital Ambroise Paré, Boulogne-Billancourt*)*; Isabelle Fredenucci, Jean-Pierre Flandrois (Centre Hospitalier Lyon-Sud, Lyon); Germany: Stefan Niemann (National Reference Centre for Mycobacteria, Forschungszentrum Borstel, Hamburg), Roland Diel (School of Public Health, University of Düsseldorf); Italy: Stefano Bonora (Università di Verona); Leonardo A Sechi, Stephania Zanetti (Università di Sassari); Carlo Garzelli (Università di Pisa); the Netherlands: Martien Borgdorff (KNCV Tuberculosis Foundation) Petra de Haas, Kristin Kremer, Dick van Soolingen (RIVM); Spain: Montserrat Ruiz, Juan Carlos Rodríguez, Gloria Royo (Universidad Miguel Hernández, Elche); Ana Pérez Meixeira, Jenaro Astray (Public Health Institute Getafe, Madrid), Juana Cacho, Amador Ramos (Hospital Universitario de Getafe); Maria Jose Iglesias (University of Zaragoza), Sofia Samper (Hospital Universitario Miguel Servet, Zaragoza); United Kingdom: Andrew Hayward, John Watson, Francis Drobniewski (Health Protection Agency, London); Jeremy Dale (University of Surrey) on behalf of the Steering Committee, Molecular Epidemiology of Tuberculosis in London; Sweden: Malin Ridell, Liselott Svensson (Institute of Medical Microbiology and Immunology, Göteborg University); Czech Republic: Milan Kubin (Institute of Hygiene of the City of Prague); Estonia: Annika Krüüner (Tartu University, Estonia, and Karolinska Institute, Stockholm, Sweden); Russia: Olga Toungoussova (University of Oslo, Norway), Dominique Caugant (Norwegian Institute of Public Health, Oslo, Norway), Andrey Mariandyshev (Northern State Medical University, Archangel): Olga Narvaskaya, Igor Mokrousov (St. Petersburg Pasteur Institute), Tatjana Otten, Boris Vyshnevskiy (Research Institute of Phthisiopulmonology, St. Petersburg); Iran: Mehrnoosh Doroudchi (Shiraz University of Medical Sciences); Cameroon: Sara Ngo Niobe-Eyangoh (Centre Pasteur du Cameroun, Yaoundé); Ethiopia: Judith Bruchfeld (Swedish Institute for Infectious Disease Control, Solna); Guinea Bissau: Tuija Koivula, Gunilla Kallenius (Swedish Institute for Infectious Disease Control, Solna); Malawi: Amelia Crampin, Judith Glynn (London School of Hygiene and Tropical Medicine, UK) on behalf of The Karonga Prevention Study (Chilumba, Malawi); South Africa: Madalene Richardson, Paul van Helden, Rob Warren, Nulda Beyers (Stellenbosch University, Cape Town); Sudan: Ghada Sharaf-Eldin (National Health Laboratory, Khartoum); Zimbabwe: Philippa Easterbrook, Shahed Murad, Francis Drobniewski (King’s College London, UK); Cuba: Raul Diaz (Instituto Pedro Kourí, Havana); United States: Barry Kreiswirth (International Center for Public Health, Newark, NJ); Midori Kato-Maeda, Elizabeth Fair, Sebastien Gagneux, Peter Small (Stanford University, Stanford, CA); Argentina: Nora Morcillo (Reference Laboratory of Buenos Aires Tuberculosis Control Program) Angel Cataldi (National Institute of Agricultural Technology); Brazil: Lucilaine Ferrazoli (Instituto Adolfo Lutz, Sao Paulo); India: Kristin Kremer (RIVM), P. Seth (All India Institute of Medical Sciences, New Delhi); Bangladesh: Leen Rigouts, Isdore Chola Shamputa (Institute of Tropical Medicine, Antwerp, Belgium); Indonesia: Reinout van Crevel (University Medical Center Nijmegen, the Netherlands); Malaysia: Jeremy Dale (University of Surrey, Guildford, UK); Thailand: Wolfgang Prodinger (Medizinische Universität Innsbruck, Austria), Porntip Bunyaratevej (Mahidol University, Bangkok); China: James Douglas (University of Hawaii); Li Weimin (Beijing Tuberculosis and Chest Tumor Institution), Kristin Kremer (RIVM); K.M. Kam (Tuberculosis Reference Laboratory, Hong Kong); Japan: Ritsuko Ohata (Okayama Prefectural Institute for Environmental Science and Public Health); Mongolia: N. Naranbat (National Center for Communicable Diseases, Ulaanbaatar); Vietnam: Dang Duc Anh (National Institute of Hygiene and Epidemiology, Hanoi); Mai Huyen, Nguyen Thi Ngoc Lan (Ho Chi Minh City); Taiwan: Ruwen Jou (Center for Disease Control, Taipei).

**Table A2:** Proportion of tuberculosis cases caused by the Beijing genotype in different studies

Study	Period	Overall Beijing/total (%)	Excluding immigrants,* Beijing/total (%)
Western Europe			
	Austria: western	1993–2004	12/895 (1.3)	7/673 (1.0)
	Denmark: entire	1992–2001	96/3,844 (2.5)	17/1,659 (1.0)
	Finland: entire	2000–2002	23/1,246 (1.9)	13/1,119 (1.2)
	France			
	Paris area	1995	3/272 (1.1)	0/91 (0.0)
	Paris	2001–2003	5/66 (7.6)	1/22 (4.6)
	Lyon	2003	3/85 (3.5)	1/31 (3.2)
	Germany: Hamburg	2001	6/178 (3.4)	1/80 (1.3)
	Italy			
	Verona	1996–1997	2/158 (1.3)	0/131 (0.0)
	Sardinia	1997–1998	2/350 (0.6)	0/28 (0.0)
	Tuscany	2002	7/245 (2.9)	2/153 (1.2)
	Netherlands: entire	1993–2002	522/8,510 (6.1)	202/3,469 (5.8)
	Spain			
	Elche	1993–1999	0/146 (0.0)	
	Madrid	1992–2001	0/507 (0.0)	0/484 (0.0)
	Zaragoza	1993–1995	2/569 (0.4)	0/544 (0.0)
	Sweden: western	1999–2002	11/212 (5.2)	3/77 (3.9)
	United Kingdom			
	Inner London	1993	18/547 (3.3)	5/164 (3.0)
	London	1995–1997	108/2,490 (4.3)	10/273 (3.7)
Central and Eastern Europe			
	Czech Republic			
	Prague	1998	2/111 (1.8)	1/104 (1.0)
	South Moravia	1998	5/120 (4.2)	0/114 (0.0)
	Estonia: entire	1994	61/209 (29.2)	61/209 (29.2)
	Russia			
	St. Petersburg	1999–2001	133/236 (56.4)	133/236 (56.4)
	Archangel	1998–1999	54/119 (45.4)	
North Africa and Middle East			
	Iran: several areas	1995–1997	10/101 (9.9)	6/81 (7.4)
Sub-Saharan Africa			
	Cameroon†: western region	1997–1998	0/456 (0.0)	0/456 (0.0)
	Ethiopia: Addis Ababa	1996	0/121 (0.0)	0/121 (0.0)
	Guinea Bissau: Bissau	1989–1993	1/229 (0.4)	1/221 (0.5)
	Malawi: Karonga District	1996–2003	44/1,030 (4.3)	38/785 (4.8)
	South Africa: Cape Town	1992–1998	140/847 (16.5)	140/847 (16.5)
	Sudan: Khartoum	1998–1999	0/49 (0.0)	0/48 (0.0)
	Zimbabwe: Harare	1997	4/214 (1.9)	4/212 (1.9)
North America			
	United States			
	New Jersey	1999	29/382 (7.6)	15/151 (9.9)
	San Francisco	1998–2000	135/492 (27.4)	12/109 (11.0)
Caribbean			
	Cuba			
	Outside Havana	1994–1995	22/160 (13.8)	22/160 (13.8)
	Havana	1997–1998	4/51 (7.8)	4/51 (7.8)
Latin America			
	Argentina: Buenos Aires	1998–2001	5/612 (0.8)	5/582 (0.9)
	Brazil: São Paulo	2000–2001	4/420 (1.0)	3/382 (0.8)
Indian subcontinent			
	India: Delhi	1995–1996	1/83 (1.2)	
	Bangladesh: Mymensingh	2000–2002	7/97 (7.2)	
Southeast Asia			
	Indonesia: Jakarta	1998–1999	32/91 (35.2)	32/91 (35.2)
	Malaysia: Sample	1993–1994	71/426 (16.7)	65/388 (16.8)
	Thailand: Bangkok	1998–2000	98/204 (48.0)	98/204 (48.0)
	Vietnam			
	Hanoi	1998–1999	37/64 (57.8)	
	Ho Chi Minh City	1998–1999	263/499 (52.7)	
	Ho Chi Minh City	1998–2000	34/75 (45.3)	34/75 (45.3)
	Tien Giang	2003	28/60 (46.7)	28/60 (46.7)
East Asia			
	China			
	Shanghai and other areas	1994–1995	40/59 (67.8)	
	Henan	2000–2001	36/52 (69.2)	36/52 (69.2)
	Hong Kong: Hong Kong	1998–1999	356/500 (71.2)	
	Japan: Okayama	2000–2002	103/142 (72.5)	103/142 (72.5)
	Mongolia: entire	1998–1999	97/168 (57.7)	97/168 (57.7)
	Taiwan: entire	2002	187/421 (44.4)	

*Immigration status not known for all patients. †The spoligotype of 1 isolate in this study had only spacers 40–43, but other genetic markers showed it to be *Mycobacterium africanum*.

**Table A3:** Drug resistance in Beijing and other genotypes of Mycobacterium tuberculosis among nonimmigrants*†

Study	n	% resistant to each drug (no.)					
		Any drug	Isoniazid	Rifampicin	Streptomycin	Ethambutol	MDR
West Europe							
Austria: western							
Beijing	7	14.3 (1)	0.0 (0)	14.3 (1)	0.0 (0)	0.0 (0)	0.0 (0)
Other	647	6.7 (43)	2.9 (19)	2.2 (14)	3.9 (25)	0.78 (5)	0.93 (6)
Denmark							
Beijing	16	12.5 (2)	12.5 (2)	6.3 (1)	12.5 (2)	6.3 (1)	6.3 (1)
Other	1,623	10.2 (165)	3.1 (50)	0.12 (2)‡	3.6 (58)	0.0 (0)§	0.0 (0)§
Finland							
Beijing	13	15.4 (2)	7.7 (1)	7.7 (1)	16.7 (2)	0.0 (0)	7.7 (1)
Other	1,102	4.6 (51)	1.5 (17)	0.27 (3)‡	1.2 (12)§	0.73 (5)	0.0 (0)‡
The Netherlands							
Beijing	199	9.1 (18)	3.5 (7)	0.50 (1)	7.0 (14)		0.50 (1)
Other	3,239	5.8 (189)	3.2 (105)	0.22 (7)	3.9 (125)‡		0.15 (5)
Western Sweden							
Beijing	3	33.3 (1)	33.3 (1)	0.0 (0)	33.3 (1)	0.0 (0)	0.0 (0)
Other	72	5.6 (4)	2.8 (2)	0.0 (0)	1.4 (1)	0.0 (0)	0.0 (0)
United Kingdom							
Inner London							
Beijing	5	0.0 (0)	0.0 (0)	0.0 (0)			0.0 (0)
Other	145	2.8 (4)	2.1 (3)	0.69 (1)			0.69 (1)
London							
Beijing	10	20.0 (2)	20.0 (2)	0.0 (0)	10.0 (1)	0.0 (0)	0.0 (0)
Other	259	11.2 (29)	9.3 (24)	3.5 (9)	4.3 (11)	1.2 (3)	3.5 (9)
Eastern Europe							
Estonia							
Beijing	61	70.5 (43)	59.0 (36)	34.4 (21)	59.0 (36)	19.7 (12)	34.4 (21)
Other	148	14.2 (21)¶	8.8 (13)¶	2.7 (4)¶	8.1 (12)¶	1.4 (2)¶	2.0 (3)¶
Russia							
St. Petersburg							
Beijing	133	90.2 (120)	74.4 (99)	67.7 (90)	86.5 (115)	12.0 (16)	60.2 (80)
Other	103	74.8 (77)§	52.4 (54)¶	47.6 (49)§	70.9 (73)§	2.9 (3)§	42.7 (44)§
Archangel#							
Beijing	54	79.6 (43)	64.8 (35)	46.3 (25)	75.9 (41)	44.4 (24)	46.3 (25)
Other	65	36.9 (24)¶	30.8 (20)¶	7.7 (5)¶	23.1 (15)¶	21.5 (14)§	7.7 (5)
Sub-Saharan Africa							
Malawi**							
Beijing	43	0.0 (0)	0.0 (0)	0.0 (0)	0.0 (0)	0.0 (0)	0.0 (0)
Other	964	6.6 (64)	6.2 (60)	0.6 (6)	5.7 (24)	0.47 (2)	0.6 (6)
United States							
San Francisco							
Beijing	12	8.3 (1)	8.3 (1)	8.3 (1)	8.3 (1)	8.3 (1)	8.3 (1)
Other	96	16.7 (16)	2.1 (2)	3.1 (3)	11.5 (11)	1.0 (1)	0.0 (0)
Caribbean							
Cuba							
Outside Havana							
Beijing	22	50.0 (11)	0.0 (0)	9.1 (2)	50.0 (11)	0.0 (0)	0.0 (0)
Other	136	6.6 (9)¶	2.9 (4)	1.5 (2)	4.4 (6)¶	0.7 (1)	1.5 (2)
Havana							
Beijing	4	50.0 (2)	0.0 (0)	0.0 (0)	50.0 (2)	0.0 (0)	0.0 (0)
Other	47	4.3 (2)‡	0.0 (0)	0.0 (0)	4.3 (2)‡	0.0 (0)	0.0 (0)
Latin America							
Argentina: Buenos Aires							
Beijing	5	0.0 (0)	0.0 (0)	0.0 (0)	0.0 (0)	0.0 (0)	0.0 (0)
Other	548	29.0 (159)	22.5 (123)	15.0 (82)	18.3 (100)	9.1 (50)	14.6 (80)
Brazil: São Paulo							
Beijing	4	50.0 (2)††	50.0 (2)	50.0 (2)	50.0 (2)	0 (0.0)	50.0 (2)
Other	416	15.1 (63)	9.9 (41)	6.3 (26)‡	3.4 (14)§	2.4 (10)	4.8 (20)‡
Indian subcontinent							
Bangladesh							
Beijing	7	71.4 (5)	71.4 (5)	28.6 (2)	42.9 (3)	28.6 (2)	28.6 (2)
Other	89	20.2 (18)§	14.6 (13)§	4.5 (4)	12.4 (11)	1.1 (1)‡	3.4 (3)‡
Southeast Asia							
Indonesia: Jakarta							
Beijing	28	35.7 (10)	35.7 (10)	7.1 (2)	14.3 (4)	3.6 (1)	7.1 (2)
Other	53	22.6 (12)	17.0 (9)	3.8 (2)	5.7 (3)	5.7 (3)	3.8 (2)
Malaysia							
Beijing	64	6.3 (4)	3.1 (2)	3.1 (2)	4.7 (3)	3.1 (2)	3.1 (2)
Other	322	14.9 (48)	8.7 (28)	3.7 (12)	8.7 (28)	2.2 (7)	3.1 (10)
Thailand: Bangkok							
Beijing	98	29.6 (29)	12.2 (12)	7.1 (7)	20.4 (20)	3.1 (3)	4.1 (4)
Other	106	31.1 (33)	5.7 (6)	5.7 (6)	18.9 (20)	6.6 (7)	0.9 (1)
Vietnam							
Hanoi#							
Beijing	33	60.6 (20)	60.6 (20)	36.4 (12)	39.4 (13)	21.2 (7)	36.4 (12)
Other	20	25.9 (7)§	22.2 (6)§	11.1 (3)‡	7.4 (2)§	3.7 (1)	7.4 (2)§
Ho Chi Minh 1#							
Beijing	81	43.2 (35)	27.2 (22)	6.2 (5)	42.0 (34)	6.2 (5)	6.2 (5)
Other	87	26.4 (23)‡	19.5 (17)	2.3 (2)	17.2 (15)¶	2.3 (2)	2.3 (2)
Ho Chi Minh 2							
Beijing	34	52.9 (18)	41.2 (14)	5.9 (2)	50.0 (17)	0.0 (0)	5.9 (2)
Other	41	34.2 (14)	22.0 (9)	4.9 (2)	24.4 (10)‡	0.0 (0)	4.9 (2)
Tien Giang							
Beijing	28	53.6 (15)	21.4 (6)	7.1 (2)	53.6 (15)	3.6 (1)	7.1 (2)
Other	32	34.4 (11)	15.6 (5)	0.0 (0)	28.1 (9)‡	0.0 (0)	0.0 (0)
East Asia							
China							
Shanghai#							
Beijing	25	44.0 (11)	28.0 (7)	16.0 (4)	30.4 (7)	0.0 (0)	16.0 (4)
Other	14	57.1 (8)	35.7 (5)	14.3 (2)	35.7 (5)	21.4 (3)‡	14.3 (2)
Henan							
Beijing	36	33.3 (12)	25.0 (9)	16.7 (6)	27.8 (10)	8.3 (3)	13.9 (5)
Other	16	31.3 (5)	18.9 (3)	6.3 (1)	25.0 (4)	0.0 (0)	6.3 (1)
Hong Kong							
Beijing	356	13.5 (48)	5.3 (19)	0.6 (2)	9.3 (33)	1.1 (4)	0.6 (2)
Other	144	18.1 (26)	8.3 (12)	0.7 (1)	13.9 (20)	1.4 (2)	0.7 (1)
Mongolia							
Beijing	97	48.5 (47)	27.8 (27)	2.1 (2)	39.2 (38)	4.1 (4)	2.1 (2)
Other	71	50.7 (36)	16.9 (12)	0.0 (0)	46.5 (33)	0.0 (0)	0.0 (0)
Taiwan#							
Beijing	181	49.7 (90)	33.7 (61)	21.0 (38)	21.0 (38)	27.1 (49)	19.3 (35)
Other	172	35.5 (61)§	24.4 (42)	15.7 (27)	19.8 (34)	16.9 (29)‡	15.7 (27)

*Studies with >3 patients with Beijing genotype tuberculosis with known drug sensitivity included. In all but 3 of the included studies, >97% of patients were tested for drug resistance. The 3 exceptions were Argentina (95%), inner London (91%), and Jakarta (79%). In some studies, not all patients were tested for every drug. In some studies, "any drug" includes drugs not shown on the table (e.g., pyrazinamide). In Zimbabwe, 0/4 patients with Beijing genotype strains had drug- resistant strains; whether other strains were resistant is unknown. †MDR, multidrug resistant, resistant to at least isoniazid and rifampin. ‡p<0.05. §p<0.01. ¶p<0.001. #Immigration status not known. **Includes immigrants from neighboring countries. ††The 2 patients with drug-resistant Beijing strains in this study were brothers.
